# Optimizing process flow diagrams to guide implementation of a colorectal cancer screening intervention in new settings

**DOI:** 10.1007/s10552-023-01769-w

**Published:** 2023-09-21

**Authors:** Meghan C. O’Leary, Kristen Hassmiller Lich, Daniel S. Reuland, Alison T. Brenner, Alexis A. Moore, Shana Ratner, Sarah A. Birken, Stephanie B. Wheeler

**Affiliations:** 1 Department of Health Policy and Management, Gillings School of Global Public Health, University of North Carolina at Chapel Hill, Chapel Hill, NC USA; 2 Lineberger Comprehensive Cancer Center, University of North Carolina at Chapel Hill, Chapel Hill, NC USA; 3 Department of Medicine, Division of General Medicine and Clinical Epidemiology, University of North Carolina School of Medicine, Chapel Hill, NC USA; 4 Center for Health Promotion and Disease Prevention, University of North Carolina at Chapel Hill, Chapel Hill, NC USA; 5 Department of Implementation Science, Wake Forest University School of Medicine, Winston-Salem, NC USA; 6 Atrium Health Wake Forest Baptist Comprehensive Cancer Center, Winston-Salem, NC USA

**Keywords:** Colorectal cancer screening, Evidence-based interventions, Process flow diagramming, Decision-making, Community health centers

## Abstract

**Purpose:**

The goal of this study was to assess acceptability of using process flow diagrams (or process maps) depicting a previously implemented evidence-based intervention (EBI) to inform the implementation of similar interventions in new settings.

**Methods:**

We developed three different versions of process maps, each visualizing the implementation of the same multicomponent colorectal cancer (CRC) screening EBI in community health centers but including varying levels of detail about how it was implemented. Interviews with community health professionals and practitioners at other sites not affiliated with this intervention were conducted. We assessed their preferences related to the map designs, their potential utility for guiding EBI implementation, and the feasibility of implementing a similar intervention in their local setting given the information available in the process maps.

**Results:**

Eleven community health representatives were interviewed. Participants were able to understand how the intervention was implemented and engage in discussions around the feasibility of implementing this type of complex intervention in their local system. Potential uses of the maps for supporting implementation included staff training, role delineation, monitoring and quality control, and adapting the components and implementation activities of the existing intervention.

**Conclusion:**

Process maps can potentially support decision-making about the adoption, implementation, and adaptation of existing EBIs in new contexts. Given the complexities involved in deciding whether and how to implement EBIs, these diagrams serve as visual, easily understood tools to inform potential future adopters of the EBI about the activities, resources, and staffing needed for implementation.

**Supplementary Information:**

The online version contains supplementary material available at 10.1007/s10552-023-01769-w.

## Background

Process flow diagramming (or process mapping) is commonly used to support quality improvement initiatives related to healthcare delivery in diverse settings [1–4]. Broadly, process maps use visualization to describe individual process steps and the order of these steps, identify who is responsible for performing each step, clarify the process scope (e.g., starting and stopping points), and distinguish branching points in the process and the possible subsequent steps [4–7]. Documented benefits of developing these maps for quality improvement include understanding local systems and their complexity, engaging others with relevant perspectives in change initiatives, designing interventions through collective problem solving, and monitoring and measuring progress [1, 2]. These maps are also considered useful tools because they are relatively inexpensive to develop and involve minimal time and training to implement, yet they can help to create shared understanding of complex systems, identify problems or gaps, and drive change [1, 2, 8].

Given these advantages of process mapping, recent studies have called for increased utilization of process mapping to support the implementation of evidence-based interventions (EBIs), as well as further alignment between the fields of quality improvement and implementation science [9–11]. Lu and colleagues (2020), for example, identified iterative process mapping as one of three critical strategies (along with people engagement and problem solving) to be used by healthcare professionals implementing interventions [10]. Leeman, et al. [9] identified potential benefits of using process mapping and other quality improvement tools within implementation science, such as generating practice-based evidence and selecting and adapting EBIs and implementation strategies, to address gaps [9]. Process mapping can also be used across phases of implementation, from pre-implementation through sustainment and dissemination of interventions [1, 12].

However, little is known about how process maps of previously implemented interventions can inform or support the implementation of similar interventions in new settings. In their systematic review of 105 studies that utilized process mapping, Antonacci et al. [1] reported that the majority of studies only used process mapping during the early intervention stages to understand the local system and did not describe any subsequent actions or uses of these tools after the maps were developed [1]. They did, however, note that these maps offer value with respect to documenting processes that can be further disseminated [1]. For example, prior research has shown that process maps depicting the general process of health interventions can aid in the targeted application of that intervention within other settings [13]. More information is needed on the extent to which more detailed maps of EBIs can be used to guide the early phases of implementation in other contexts.

Our goal was to understand how to optimize process mapping as a tool for guiding the implementation and spread of previously implemented interventions in new sites. Using an example related to colorectal cancer (*CRC*) screening, we conducted interviews with community health professionals and practitioners, during which we presented different versions of process maps describing intervention implementation. We wanted to understand how reviewing process maps of an existing intervention can aid other healthcare teams in considering possible adoption and implementation of the intervention in their own setting, as well as the characteristics and potential uses of the process maps that facilitate decision-making about EBI implementation.

## Methods

### CRC screening intervention

**Fig. 1:**
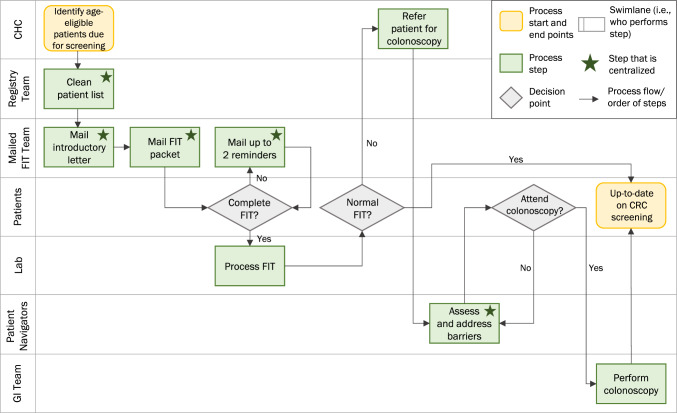
Process Flow Diagram of the SCORE Intervention (“Low Detail” Version)

In order to understand how process mapping can inform intervention implementation in new sites, we built on an existing implementation study—Scaling Colorectal Cancer Screening Through Outreach, Referral, and Engagement (SCORE). As described previously [14, 15], the multicomponent SCORE intervention included the development of a registry to identify patients eligible and due for CRC screening, mailed fecal immunochemical testing (FIT) outreach, and navigation of patients with an abnormal FIT to follow-up colonoscopy. Many of the implementation activities were performed by a centralized, offsite outreach team working in collaboration with community health centers (CHCs) to improve CRC screening while limiting the burden placed on clinical staff. Figure 1 provides an overview of the process used to implement the SCORE intervention in two CHC settings in North Carolina.

For this sub-study focused on process maps as EBI implementation tools, we used SCORE as the specific example for two primary reasons. First, one of the goals of the parent SCORE study was to determine how this type of centralized multicomponent intervention could be sustained and scaled up to reach additional sites and patients. Second, we used process mapping heavily across all phases of implementing the SCORE intervention [12, 15]. Prior to SCORE implementation, sessions with working partners, including CHC clinical and administrative staff, study leadership, quality improvement staff, and centralized outreach team members, were used to design the intervention process steps and delineate roles through the creation and iteration of process maps. SCORE researchers also used these process maps to select measures (e.g., implementation costs, fidelity, etc.) to evaluate during implementation, to develop tools for estimating those measures, and then to track outcomes for individual process steps [12]. Now that SCORE is in the sustainment planning phase, the process maps are being used to consider the feasibility of continuing and expanding this intervention.

Both this sub-study and the SCORE parent study were conducted as part of the NCI-funded consortium The Accelerating Colorectal Cancer Screening and Follow-up through Implementation Science (*ACCSIS*) Program. The overall aim of ACCSIS is to conduct multi-site, coordinated, transdisciplinary research to evaluate and improve CRC screening processes using implementation science. This sub-study was reviewed and exempted by the Institutional Review Board at the University of North Carolina at Chapel Hill.

### Process maps

During the process mapping sessions conducted prior to implementing SCORE, we developed detailed swimlane process flow diagrams for each of the core intervention components—registry development, mailed FIT outreach, and patient navigation. The swimlanes, or rows in the process map, identified the individuals and partners involved and which process steps each would perform [5]. Swimlane diagrams are an effective approach for health interventions involving care coordination across multiple groups and have previously been applied to patient navigation [16].

For this sub-study, we integrated the detailed swimlane diagrams for each intervention component into a single process map of the complete SCORE intervention. Individuals involved in performing the process steps were asked to review and identify any process changes that occurred during the implementation period (for example, due to feasibility or contextual changes). We then created three different versions of the swimlane diagram, each depicting the complete SCORE intervention but in varying levels of detail. These three versions included: 1) broad overview of SCORE depicting the core components (“Low Detail”), 2) more detailed description of SCORE that included the specific implementation activities performed for each component, such as the steps involved in mailing FIT kits and reminder letters to patients (“Medium Detail”), and 3) most detailed description of SCORE that built in the communications occurring across swimlanes, the materials used (e.g., types of documents included in mailed FIT kits), and the time periods that occurred between implementation activities (“High Detail”). Figure 1 presents the Low Detail swimlane diagram. Appendices A and B present the Medium Detail and High Detail swimlane diagrams, respectively. All versions were developed using Microsoft PowerPoint so that the process maps could be easily viewed by participants during virtual interviews. Since the Medium Detail and High Detail map versions included more granular process steps, these maps extended across multiple PowerPoint slides, as shown in the Appendix.

### Rationale

We considered contingency theory and complexity theory within organizational theory when framing our research question and designing the process maps and interview guide. Contingency theory explains that optimal intervention implementation depends on a given health organization’s internal and external characteristics [17, 18]. Optimal EBIs will differ across organizations, and the extent to which the interventions should be more or less programmed (e.g., standardized) depends on the level of uncertainty about how to implement the intervention in the particular environment. In our interviews, we were interested in how review of the SCORE process maps might allow health professionals to identify areas of possible uncertainty related to implementation and consider how to potentially adapt SCORE to their local internal and external context.

Complexity theory is used to understand complex systems in terms of system boundaries (e.g., where the intervention starts and stops), interconnections between components (e.g., roles, responsibilities, hand-offs), and characteristics of interconnections (e.g., delays, how responsibilities align with broader stakeholder objectives, goals, and values) [19–21]. Complexity theory is particularly relevant in this case as we are studying a complex intervention that is embedded in real-world community settings and involving system actors who cross organizational boundaries and disciplines that interact dynamically and affect the larger system. While process maps organize process steps involved in intervention implementation linearly, they clarify system characteristics that help community health teams understand the intervention *as a system*. We hypothesized that process mapping as a tool could be used to facilitate collective sense-making among other health teams, during which they consider how intervention implementation would modify their existing system and how they could potentially adapt the existing intervention to best fit their system.

### Interviews

We used semi-structured and cognitive interviewing to understand how community health professionals at non-SCORE sites could potentially use the process maps to inform implementation of a SCORE-like intervention in their local settings. The interviews, which were conducted virtually and lasted up to one hour, included presentation of the three process map versions. A standardized script was used to described the basic components of process maps and then walk through the process steps for each SCORE map, beginning with the Low Detail map and concluding with the High Detail map. For each map iteration, we assessed how easily participants understood both the process map and how SCORE was implemented using the process map. Participants were also asked which map characteristics they liked and disliked, what new information they learned from each map, and which questions, if any, they still had about how SCORE was implemented. After viewing all three versions, participants were asked about the utility of these maps related to EBI implementation, how the maps could be optimized to support EBI implementation, the feasibility of implementing a SCORE-like intervention in their local setting and any necessary adaptations. Sample questions included: “did these diagrams resonate with you (why/why not)?” and, “are there particular circumstances in which one or more of these diagrams would be useful to you or your organization?”

We piloted the interview guide with four individuals with backgrounds in primary care, gastroenterology, and/or quality improvement, all of whom bring substantial experience with CRC screening interventions implemented in diverse populations. Two participants were members of the SCORE team, while the other two were not familiar with SCORE. Their feedback was used to update the interview guide and process maps, for example, by animating and walking through each individual process step during the interviews (rather than asking participants to review each map independently before responding to the interview questions). We also added context about the process maps upfront in the guide, included transition slides between maps to better distinguish the different versions, and revised the wording of some process steps for clarity.

Individuals were eligible to participate in interviews if they were community health professionals or practitioners (e.g., health administrators, providers, pharmacists, quality improvement staff) at non-SCORE sites interested in improving CRC screening at their site. We identified participants through our network contacts within the Cancer Prevention and Control Research Network (CPCRN) and related networks and through collaborating center project partners. We sent email invitations to potential participants identified through these networks, and we also received direct email inquiries from community health professionals who had been directly referred by network partners. Interviewees were also asked to recommend other potential participants. Interviews were conducted until we reached saturation of themes. Participants received a $100 gift card.

### Analysis

After audio-recording and transcribing the interviews, we used rapid qualitative analysis, a rigorous, but less time-intensive, analytic approach previously used in implementation research to address targeted, action-oriented questions [22, 23]. We designed a template in Microsoft Excel for summarizing the information learned from each transcript across a series of domains including comprehension (e.g., questions about process mapping or SCORE implementation), preferences (e.g., preferred map characteristics, overall map preference), recommendations (e.g., clarifications needed about unclear process steps, map design suggestions), potential users (i.e., individuals or groups that should or could use the process maps related to EBI implementation), and acceptability/perceived utility of the process maps (e.g., specific ways that process maps would be beneficial to EBI implementation). This approach was selected because the interview guide was developed to include a list of questions to help assess each process map version (for example, what parts of this process map did participants like/dislike) and to understand what would be needed to optimize the SCORE process maps if our intent is to support spread of a SCORE-like intervention. We assessed frequency of responses by theme and identified illustrative quotes.

## Results

Eleven community health practitioners and professionals unaffiliated with SCORE completed interviews between July and September 2022 (Table 1). Most were interviewed individually, though one group interview including three participants was conducted. Participants included medical directors, nurses, case managers, pharmacists, and quality specialists. Approximately one-third served in a health administrator or leadership role at their CHC, and two-thirds had a role related to quality improvement (e.g., case manager, quality specialist, etc.). Participants were mixed in terms of their years of experience in their current roles (27% reported less than 3 years, 36% reported 3 years to less than 5 years, and 36% reported 5 years or more).

**Table 1:** Characteristics of participants interviewed about process flow diagrams

Participant characteristic	*N* (%)
Overall	11 (100)
Leadership/administrative position	
Yes	4 (36)
No	7 (64)
Current role(s)^a^	
Case managers	3 (27)
Health administrators	4 (36)
Nurses	3 (27)
Pharmacists	3 (27)
Quality specialists	3 (27)
Number of years in current role +	
Less than 3 years	3 (27)
3 years to less than 5 years	4 (36)
5 years or more	4 (36)
Region served by health system	
Eastern U.S	6 (55)
Western U.S	5 (45)

^a^Some participants have multiple roles in their current position and, thus, the percentages sum to greater than 100

+ Participants may have additional years of experience at their current site or more generally as a provider in the healthcare field

All participants reported being able to understand the Low Detail process map following the presentation. Nearly all agreed that the layout of this first map version was clean, organized, and generally easy to follow. Only one participant noted that there may have been some initial confusion about how to interpret this map in the absence of the animation (e.g., step-by-step walk through of the process map). Generally, participants found the use of swimlanes to be useful in facilitating their understanding of the process. One participant explained that swimlanes “very concretely assigned duties to specific people, so there’s not much pointing fingers and, you know, everyone has a clear identified role, what their part is to achieve the overall goal.” Participants agreed that the included decision points (e.g., branching points; FIT completion, FIT result, and colonoscopy completion) were the appropriate outcomes to assess for this type of intervention and reflected their local goals related to CRC screening. A few participants recommended adding a decision node to this Low Detail map to depict evaluation of the colonoscopy results and completion of any necessary follow-up.

Participants’ reflections after viewing only the Low Detail process map demonstrated that community health professionals at other sites are able to initiate detailed discussions about possible implementation of the SCORE intervention using this high-level swimlane diagram alone. Without prompting, participants were able to describe aspects of the SCORE intervention that they found acceptable or likely to support CRC screening, and identify other elements that would need to be adapted for their local setting. With respect to elements of the intervention that they felt were important to improve screening, participants identified mailing up to two reminders after the FIT mailing (e.g., having repeat outreach attempts), as well as having a navigator assess and address patients’ barriers to follow-up colonoscopy.

Participants’ recommendations for how the SCORE intervention should be adapted included providing patients with a choice between screening modalities upfront before initiating mailed FIT outreach and/or using a primarily Cologuard-based stool DNA testing approach to CRC screening which can also serve as an at-home test promoted through mailed outreach. Another possible adaptation was providing a more personalized approach to the initial screening process through use of a phone call in place of or in addition to either the introductory letter or reminder letter offered through SCORE. Participants also described a need to develop an accurate and efficient system for ensuring that all follow-up colonoscopy records, including the test results and surveillance recommendations, are documented in their local electronic health records. Although the SCORE program does include process steps in the more detailed map versions to ensure that providers and patients are notified of the colonoscopy results, these respondents felt this was a critical component that should be further emphasized and developed to address local challenges with their CRC screening programs.

Individuals interviewed reflected on the additional details provided about the implementation of the SCORE intervention after viewing the Medium Detail and High Detail maps. Participants had mixed feedback regarding how much detail is too much information, with some describing one or both of these map versions as too busy and others appreciating the additional information regarding implementation. Despite these differences in their overall impressions of the length of these maps and quantity of information provided, participants generally agreed on the types of added information that were useful versus those that were unnecessary to capture. Participants recommended adding details about the full scope of navigation services, the activities performed after FITs are determined to be invalid, the methods for how patient interactions and results are tracked, and the time intervals between attempts to reach patients about screening. In contrast, participants recommended excluding or condensing the level of detail provided about the specific activities involved in conducting mailed FIT outreach.

Across the process map versions, individuals interviewed identified multiple opportunities for using the process maps to support or inform implementation of a similar intervention (Table 2). Perceived utility of these types of tools for EBI implementation included *planning for implementation* by identifying adaptations needed to fit the local system; *role delineation* and development of standardized workflows; *training* clinic staff and other system actors on how to implement the intervention; *quality control* including monitoring patient progress through the system, confirming that system actors performed their assigned tasks, and addressing problems as needed; and *securing buy-in* for intervention implementation from a range of community health professionals including clinicians and leadership.

**Table 2:** Themes regarding perceived utility of process maps for informing intervention implementation

Theme	Theme description	# Respondents	Illustrative quote
Planning for implementation	Modifying the existing process map template for the local context	11	“[It] would take reinventing the wheel out [because] if [quality improvement teams] have to do all the planning…basically it would take another six to eight months.”
Role delineation	Understanding the roles and responsibilities of individuals involved in implementation and developing standardized workflows	9	“This would be a step-by-step workflow which is for any organization mandatory. You need workflows. People can’t just be doing whatever they want, or whatever they feel like. And so there's accountability.”
Training	Training staff on how to implement the intervention and/or clarifying the intervention at clinic meetings	7	“I think that level of detail [for the High Detail map] to me makes the most sense at like an initial meeting where we're going over—what is this project? How is it going to impact your clinic? Who's doing what? And then I also see it used in like a training session. So like I'm training a new employee who [would] champion it for our department…they know from A to Z what their part is, but also how their part impacts other people's parts.”
Quality control	Monitoring patients’ progress through the system and ensuring all steps are carried out as intended	6	“...the main factor with anything like this is ensuring, one, quality control and then that factor of making sure that there's no errors. It always helps with that when things are detailed and outlined.”
Securing buy-in	Obtaining support for intervention implementation from community health leadership and staff	2	“If I'm working with the clinical providers and the clinical teams, I would [use the Medium or High Detail version] so that they can see that all of the I’s are dotted and the T's are crossed, and so that they don’t ask questions, because for them it's all about liability and making sure that everything is taken care of.”

Participants described how they would use the different process map versions for distinct purposes, most commonly comparing the Low Detail and High Detail process maps. With respect to the Low Detail process map, participants described it as more of a template that can be adapted for individual clinics. Relative to the High Detail map, one participant explained the Low Detail map “was a little more flexible for us to be able to kind of merge together and take some steps out” to meet the staffing structure and resources of their own site. Another theme was the ability to more easily share and disseminate the Low Detail map as a handout or overview to staff during meetings (but then verbally present on either of the more detailed map versions). One health professional explained: “I think the [Low Detail map] was very simple, straightforward, and if I was going through the steps with somebody, you know, I would be more detailed. The chart wouldn’t be that detailed, but the discussion would be more detailed.” Participants identified different potential users of the Low Detail map. Some felt the target users were staff and leadership who are less involved with CRC screening and only need to know high-level information (and not the detailed implementation steps). Others identified the possible users as staff who are heavily involved in quality improvement efforts and, thus, likely able to quickly infer what is needed for each of the high-level process steps without needing the more detailed versions.

Regarding the High Detail process map, community health professionals described using this type of process map when planning out the intervention for their respective teams. Participants noted that the additional details would be important when training or re-training staff on implementation tasks or for staff to have as a reference point when completing their individual tasks. Another perceived use of this more detailed map was to create accountability for tasks within teams to support monitoring and quality control. Respondents discussed sharing this map version with their team as a specific example of how this intervention was implemented previously, but noted that it may be more difficult to adapt for the local context compared to the Low Detail map. As with the Low Detail map, participants differed in terms of the audiences they felt would find the High Detail version most useful. This could be the clinic and community health staff most involved in CRC screening initiatives who would need the additional information or, conversely, the clinical providers and staff from whom they are trying to obtain buy-in for adopting the intervention.

## Discussion

This study provided early evidence of the acceptability of using process flow diagrams to inform future adoption and implementation of an existing intervention in new contexts. We found that community health professionals and practitioners who were previously unfamiliar with the SCORE intervention were able to use SCORE process maps to quickly understand how the intervention was implemented and initiate discussion around the feasibility of adopting the intervention and the adaptations required for their local context.

Individuals interviewed identified several characteristics of process maps that were most conducive to understanding how the SCORE intervention was implemented. Participants agreed that the inclusion of swimlanes was useful for understanding role delineation and provided an organized layout for following the process steps. They noted the relevancy of the decision/branching points included in the process maps to their own health system’s goals regarding CRC screening. Participants also reported that, in addition to using these process maps as standalone diagrams, the animation and guided walk through of each map helped to facilitate their understanding of the process map and how the intervention was implemented.

The study findings echoed prior research describing how process maps can be used as a type of tool or implementation strategy to support the adoption, planning, and implementation of EBIs [1, 10, 11]. Participants reflected on opportunities to use the SCORE intervention process maps as a type of template that can be modified to the local system. Use of the Low Detail process map, in particular, was viewed as a more generic overview to guide the key implementation activities in their setting. They also described potential uses of these maps in terms of training staff on implementation, obtaining buy-in or support from other health professionals at their sites, standardizing workflows, and creating accountability and built-in monitoring opportunities during implementation—all activities that are iterative and can occur across different phases of implementation.

We found that participants were able to reflect on the similarities and differences between the context in which the SCORE intervention was implemented and their local organization’s characteristics, resources, and needs. Guided by contingency theory, we observed that community health professionals were using the maps to query and explore possible uncertainties at every point (e.g., process step) in the map and how those uncertainties could impact implementation. As one example, participants described the extent to which their community health site had the types of staff who could perform each swimlane; if it was determined that a single staff person would need to be responsible for tasks in multiple swimlane from the SCORE process map, there was less certainty about how the existing intervention would fit their local context. Similarly, participants had difficulty in considering how SCORE’s centralized model would translate to their own site (e.g., which individuals or organizations would be the centralized entity in their environment) and, thus, less certainty about how SCORE could guide implementation. There were other scenarios in which participants used the maps to identify needed adaptations—such as utilizing more phone-based outreach compared to mailed outreach to meet the particular needs of their local patient populations. In these cases, we observed that being able to provide people with an existing model can prompt them to explore ideas for how to complete a complex task.

There are a number of complexity-aware methods that can be used to understand complex systems and describe how complex interventions are intended to work. In this study, we found process flow diagrams to be one such useful tool because they helped community health professionals to identify what needed to be done and in what order across organizational and role boundaries, and served as a foundation for assessing potential delays and resource adequacy at each process step. These maps were relatively simple to create and use, despite system complexity, as they allowed for documentation of complex interactions across entities. We found that system actors were able to understand and build on maps to explain how a process would change in their setting. Process mapping allowed participants to develop a common understanding of the system in which this intervention was embedded, providing a roadmap to better discuss and brainstorm about how to address specific aspects of complexity.

Other types of complexity-aware methods can help teams illuminate different aspects of system complexity and be synergistic with process mapping. Causal loop diagrams, as one example, focus attention on ripple effects of change and ask how different system agents will respond to changes in ways that might reinforce or limit/counteract them [24, 25]. Causal loop diagrams can better explore the theory of change for a specific intervention, documenting how and why it is expected to work while probing for potential undesired consequences that might undermine its impact. In terms of synergy, our process maps captured desired intervention implementation tasks and can be used to identify leverage points, or those factors affecting the ability to complete the desired tasks, and causal loop diagrams can help to document how to build on those leverage points. As another example, the circle of care modeling approach is an example of a soft systems method that provides a patient-centric deep dive into specific aspects of connections between system components such as cross-agent communications and data flows [26]. Where possible, we recommend integrating multiple types of tools to support EBI implementation as they each provide lenses for understanding complexity; this study helped to illustrate how process maps are one acceptable tool to initiate these discussions. While more linear than some other tools, these maps help agents naturally identity some of the key considerations for possible adoption, such as what are the desired outcomes of interest (e.g., the decision nodes), which steps must be done, and who could possibly perform those steps given the activities and resources required.

Although differences were found with respect to the preferred version of the SCORE process maps and the ideal level of detail to be included, participants generally agreed that the process map versions could be used to achieve different objectives. Their feedback largely pointed towards excluding the Medium Detail version of the map and, instead, optimizing the Low Detail and High Detail maps to achieve distinct purposes. In particular, respondents identified advantages of having a simple map to build high-level understanding of the intervention and foster discussion about possible adaptations among diverse staff positions. They also discussed the value of having a more detailed map, especially for training and monitoring purposes. These potential uses highlight opportunities for health professionals to utilize different versions of the process maps to support capacity building in their local contexts.

Our study has several important limitations. First, our study assessed clinic staff perceptions of how process maps might be useful for intervention implementation, which may differ from how they would actually use these tools in practice. Second, we presented three different process map versions, and were unable to assess perceptions of other possible iterations of these diagrams. Moreover, we were not able to compare the acceptability of using process maps versus another type of quality improvement tool such as a standardized work document to inform EBI implementation. Third, a small number of health professionals were interviewed, and, nearly all of them reported extensive experience with quality improvement efforts designed to support CRC screening. Thus, we were unable to determine how health professionals who are new to CRC screening initiatives would perceive and value these process maps. By reaching those with more limited experience in this area, we could consider additional questions such as any possible unintended consequences of using process maps that are too prescribed.

This study provided insight into the ability to share our SCORE process flow diagrams with other health organizations interested in improving their local CRC screening rates as a method of initiating discussions about possible implementation of a similar intervention. Our findings showed that there is potential to use this type of quality improvement tool to support implementation across diverse settings. Future work should evaluate preferences for different process map designs, particularly in the context of complex intervention implementation, in a broader sample of health professionals. Additionally, it will be important to test and compare process maps that illustrate centralized services (as we have done here) versus embedded activities. We hope that these types of tools can help to inform potential future adopters of complex EBIs about what to expect with implementation and support their decision-making.

### Supplementary Information

Below is the link to the electronic supplementary material.

Supplementary file1 (DOCX 3296 KB)

## Data Availability

The datasets analyzed during the current study are available from the corresponding author on reasonable request.

## References

[1] Antonacci G (2021). Process mapping in healthcare: a systematic review. BMC Health Serv Res.

[2] Antonacci G (2018). The use of process mapping in healthcare quality improvement projects. Health Serv Manage Res.

[3] Kononowech J (2020). Visual process maps to support implementation efforts: a case example. Implement Sci Commun.

[4] Marriott RD (2018). Process mapping—the foundation for effective quality improvement. Curr Probl Pediatr Adolesc Health Care.

[5] Damelio R (2011). The basics of process mapping.

[6] Madison D (2005) Process mapping, process improvement, and process management: a practical guide for enhancing work and information flow. Paton Professional.

[7] SaferPak (2006) Basic tools for process improvement. http://saferpak.com/flowchart_articles/howto_flowchart.pdf. Last accessed 18 Sep 2023

[8] Durski KN (2020). Systems thinking for health emergencies: use of process mapping during outbreak response. BMJ Glob Health.

[9] Leeman J (2021). Aligning implementation science with improvement practice: a call to action. Implement Sci Commun.

[10] Lu AD (2021). Implementation strategies for frontline healthcare professionals: people, process mapping, and problem solving. J Gen Intern Med.

[11] McCreight MS (2019). Practical use of process mapping to guide implementation of a care coordination program for rural veterans. J Gen Intern Med.

[12] O’Leary MC (2022). Extending analytic methods for economic evaluation in implementation science. Implement Sci.

[13] Weir NM (2018). Application of process mapping to understand integration of high risk medicine care bundles within community pharmacy practice. Res Social Adm Pharm.

[14] Malo TL (2021). Centralized colorectal cancer screening outreach and patient navigation for vulnerable populations in North Carolina: study protocol for the SCORE randomized controlled trial. Implement Sci Commun.

[15] Ferrari RM, Leeman J, Brenner AT, et al (2023) Implementation strategies in the exploration and preparation phases of a colorectal cancer screening intervention in community health centers. Implement Sci Commun. 10.1186/s43058-023-00485-5 10.1186/s43058-023-00485-5 PMC10512568 37730659

[16] Casanova NL (2022). Development of a workflow process mapping protocol to inform the implementation of regional patient navigation programs in breast oncology. Cancer.

[17] Birken SA (2017). Organizational theory for dissemination and implementation research. Implement Sci.

[18] Leeman J (2019). Advancing the use of organization theory in implementation science. Prev Med.

[19] Lanham HJ (2013). How complexity science can inform scale-up and spread in health care: understanding the role of self-organization in variation across local contexts. Soc Sci Med.

[20] Weick KE (1995). Sensemaking in Organizations.

[21] Leeman J (2022). Applying theory to explain the influence of factors external to an organization on the implementation of an evidence-based intervention. Front Health Serv.

[22] Lewinski AA (2021). Applied rapid qualitative analysis to develop a contextually appropriate intervention and increase the likelihood of uptake. Med Care.

[23] Nevedal AL (2021). Rapid versus traditional qualitative analysis using the consolidated framework for implementation research (CFIR). Implement Sci.

[24] Baugh Littlejohns L (2021). Diverse approaches to creating and using causal loop diagrams in public health research: recommendations from a scoping review. Public Health Rev.

[25] Mills SD (2023). Using systems science to advance health equity in tobacco control: a causal loop diagram of smoking. Tob Control.

[26] Price M (2016). Circle of care modelling: an approach to assist in reasoning about healthcare change using a patient-centric system. BMC Health Serv Res.

