# Supplementary material for: Optimizing process flow diagrams to guide implementation of a colorectal cancer screening intervention in new settings
Source: Cancer Causes Control. 2023 Sep 21;34(Suppl 1):89–98. doi: 10.1007/s10552-023-01769-w (PMC10689519; doi:10.1007/s10552-023-01769-w)

Appendix A. Process Flow Diagram of the SCORE Intervention (“Medium Detail” Version)


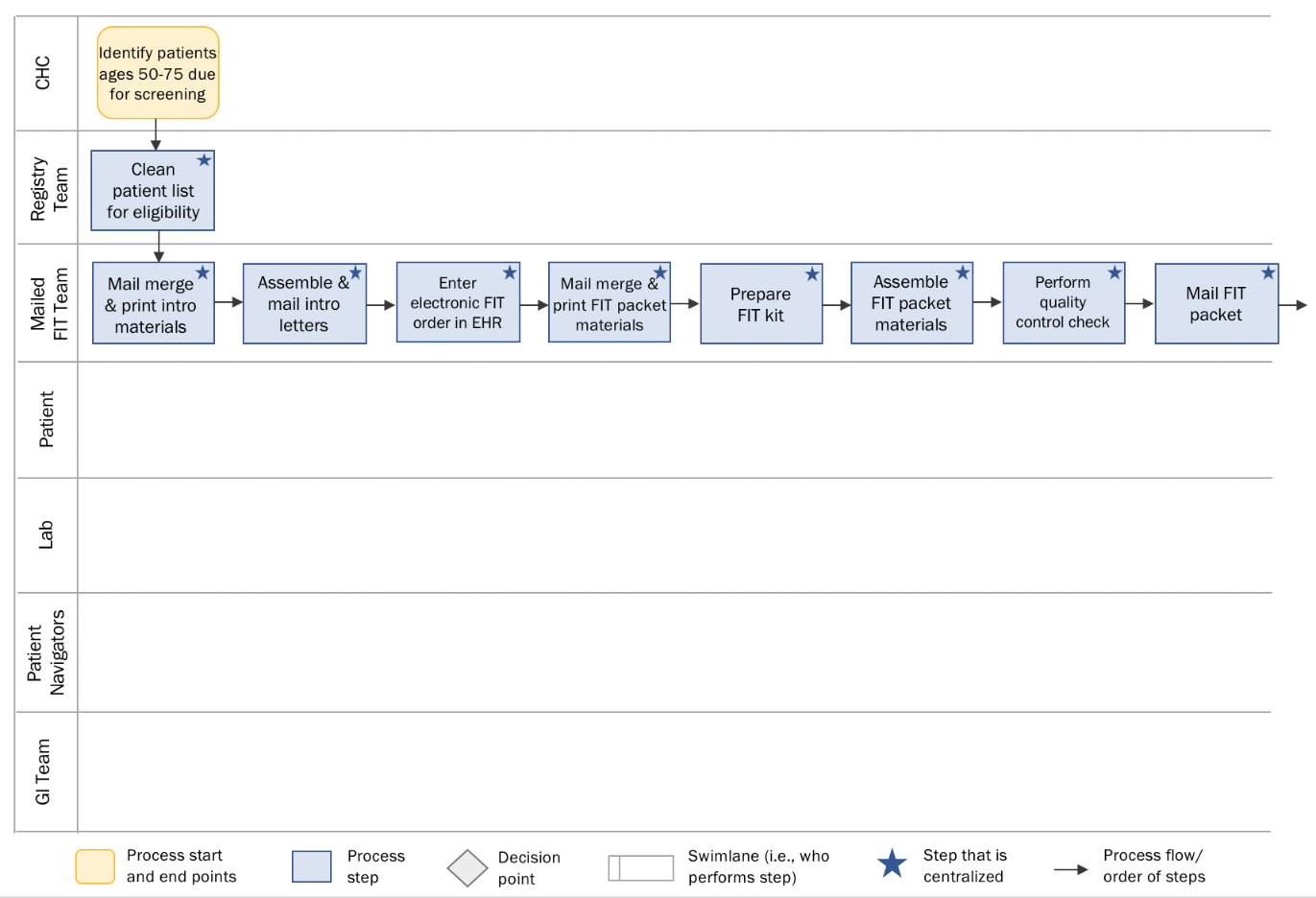


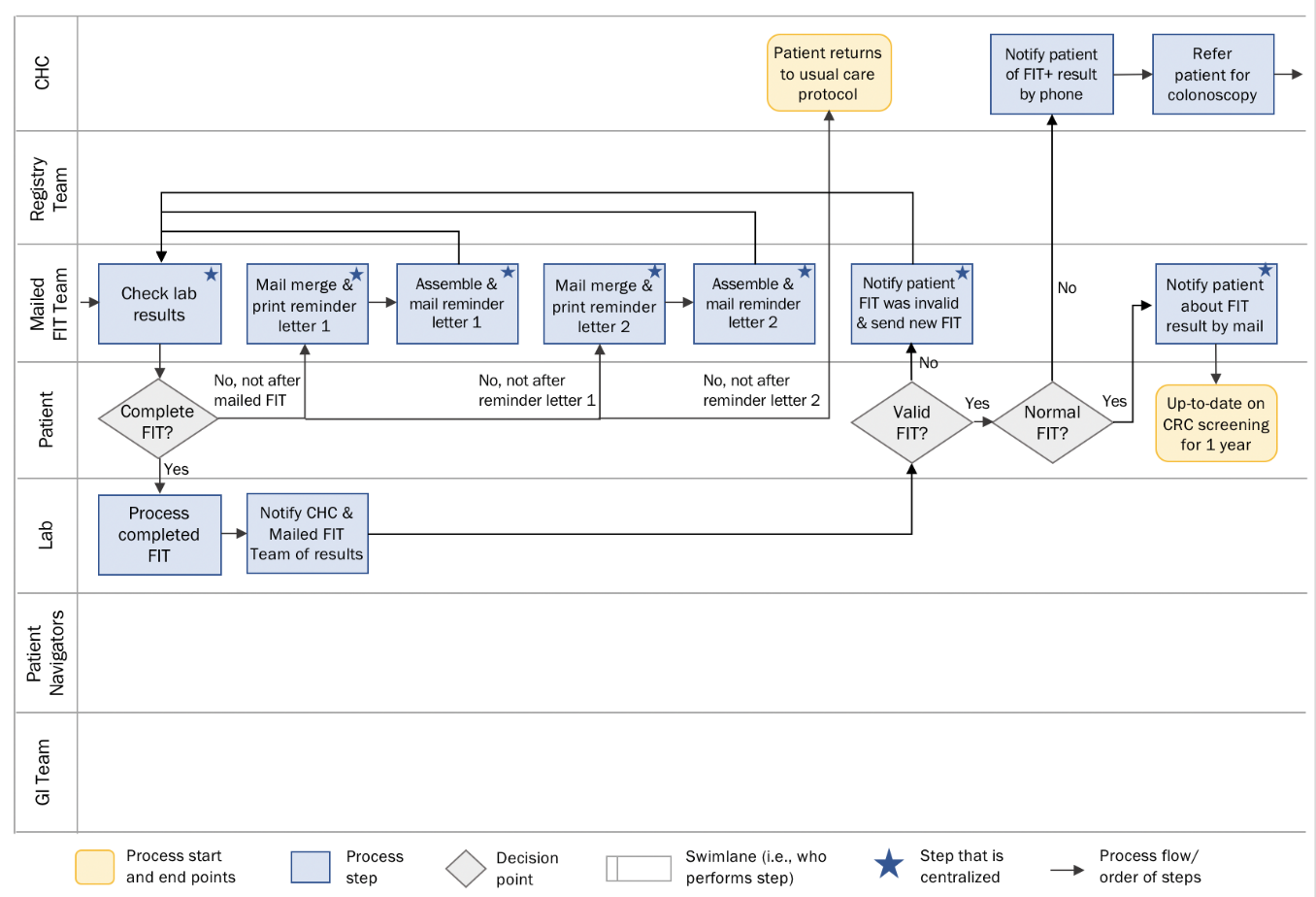


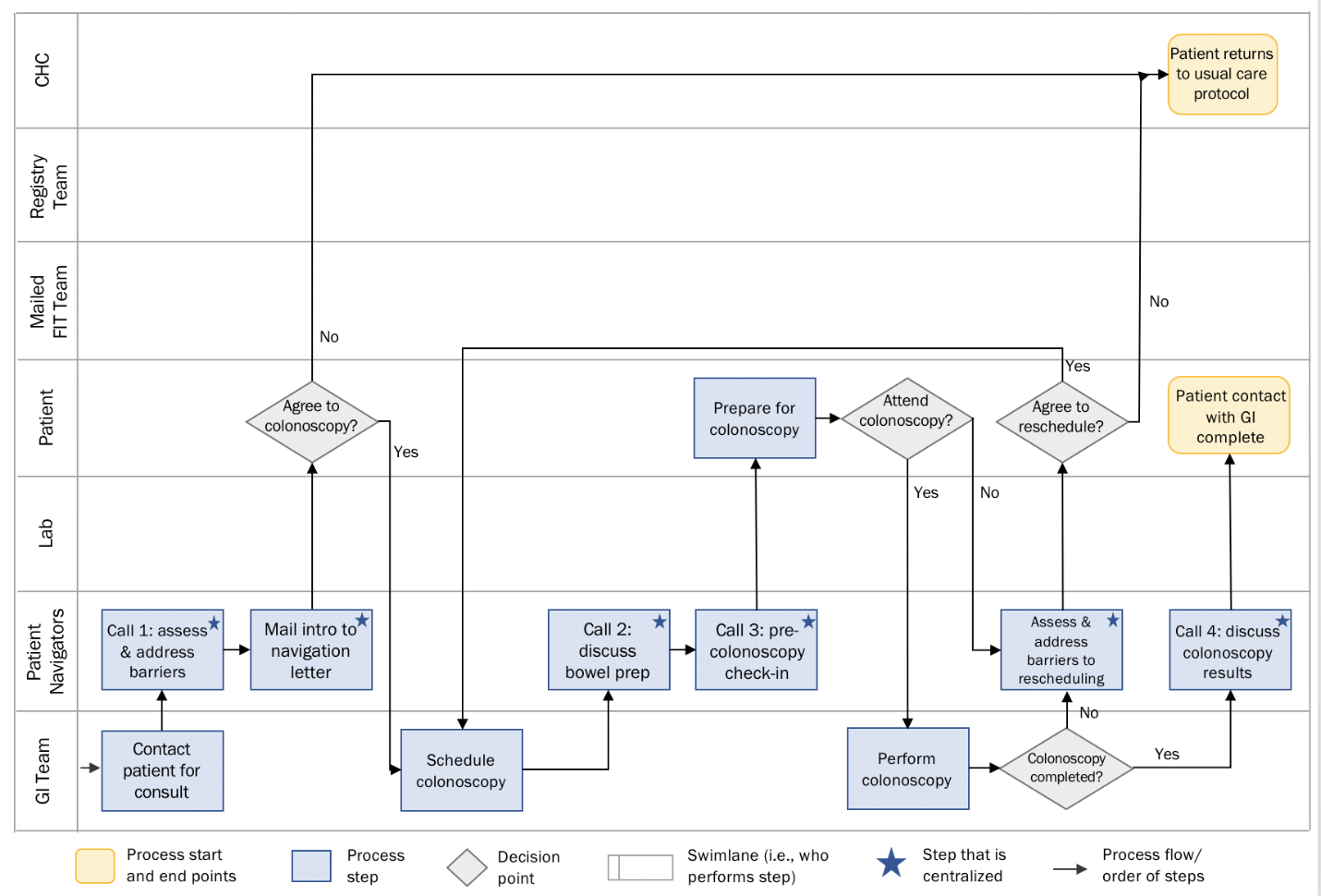


Appendix B. Process Flow Diagram of the SCORE Intervention (“High Detail” Version)


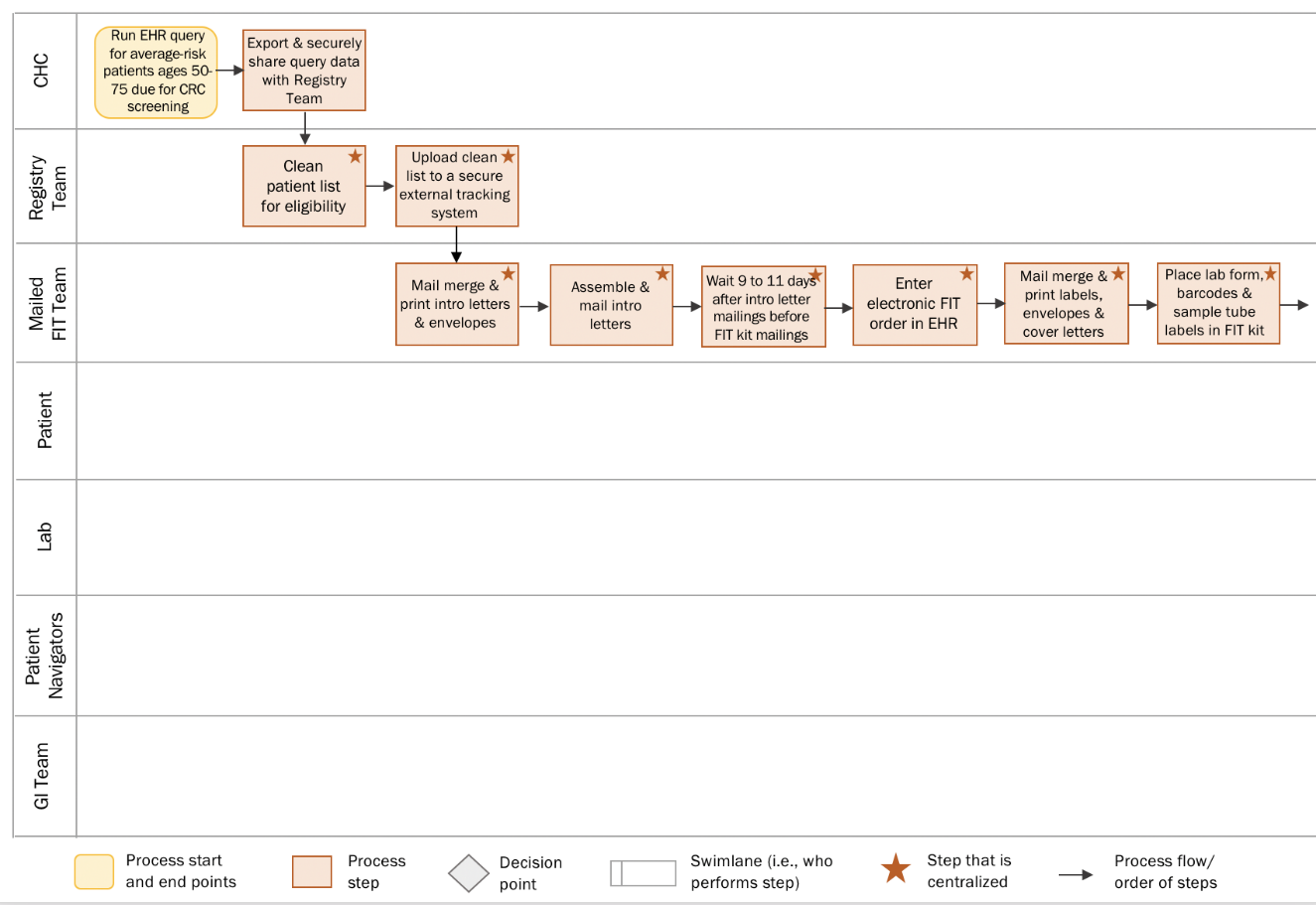


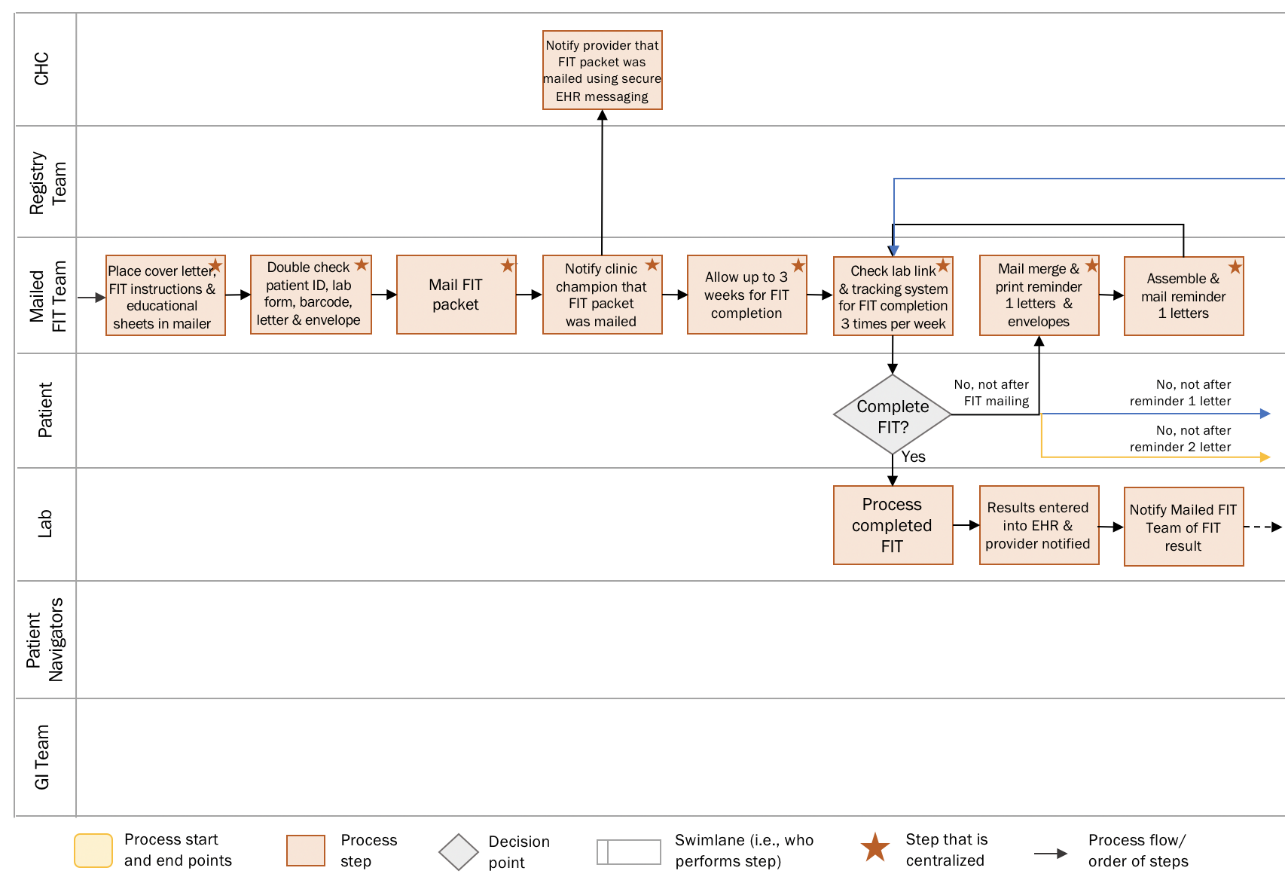


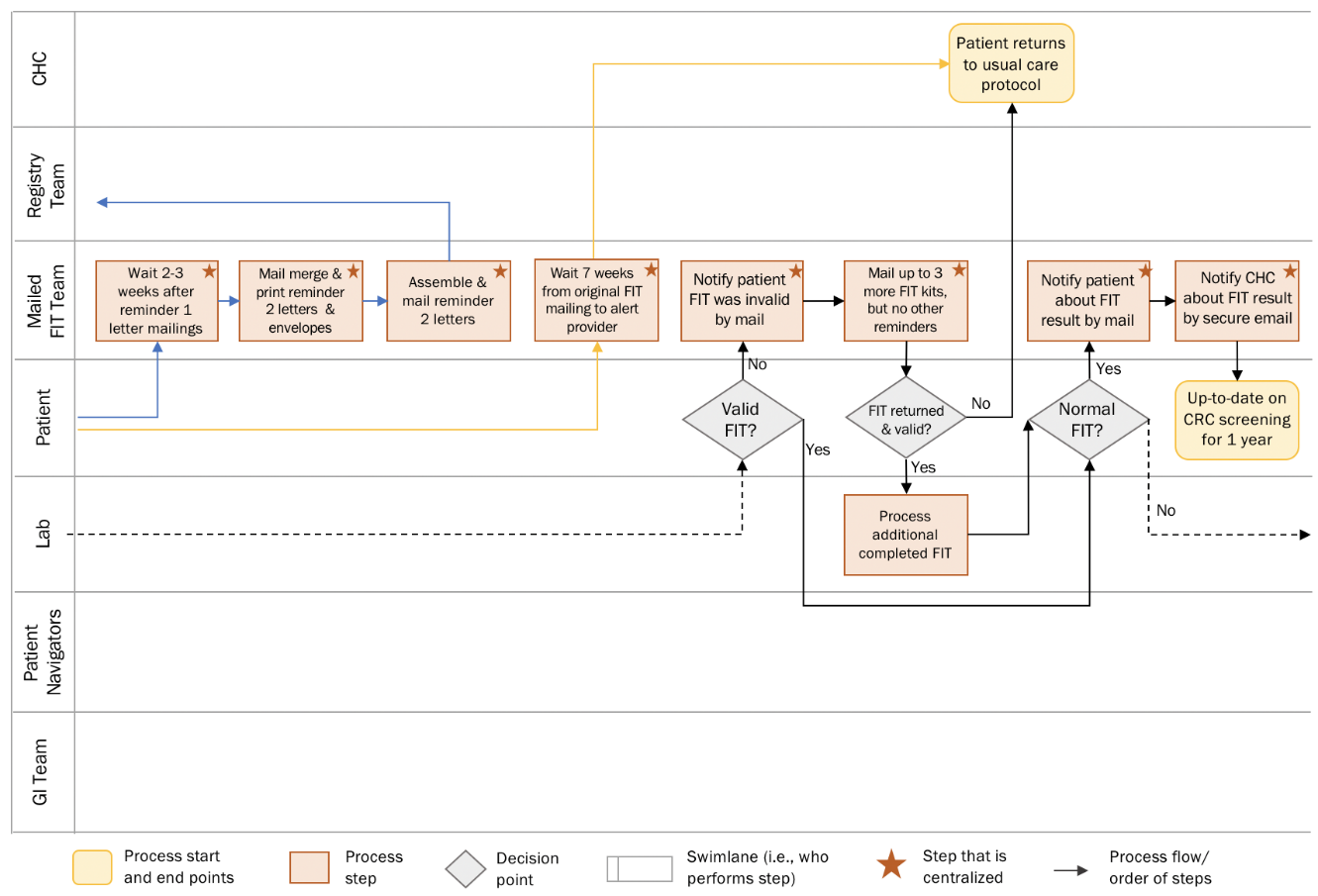

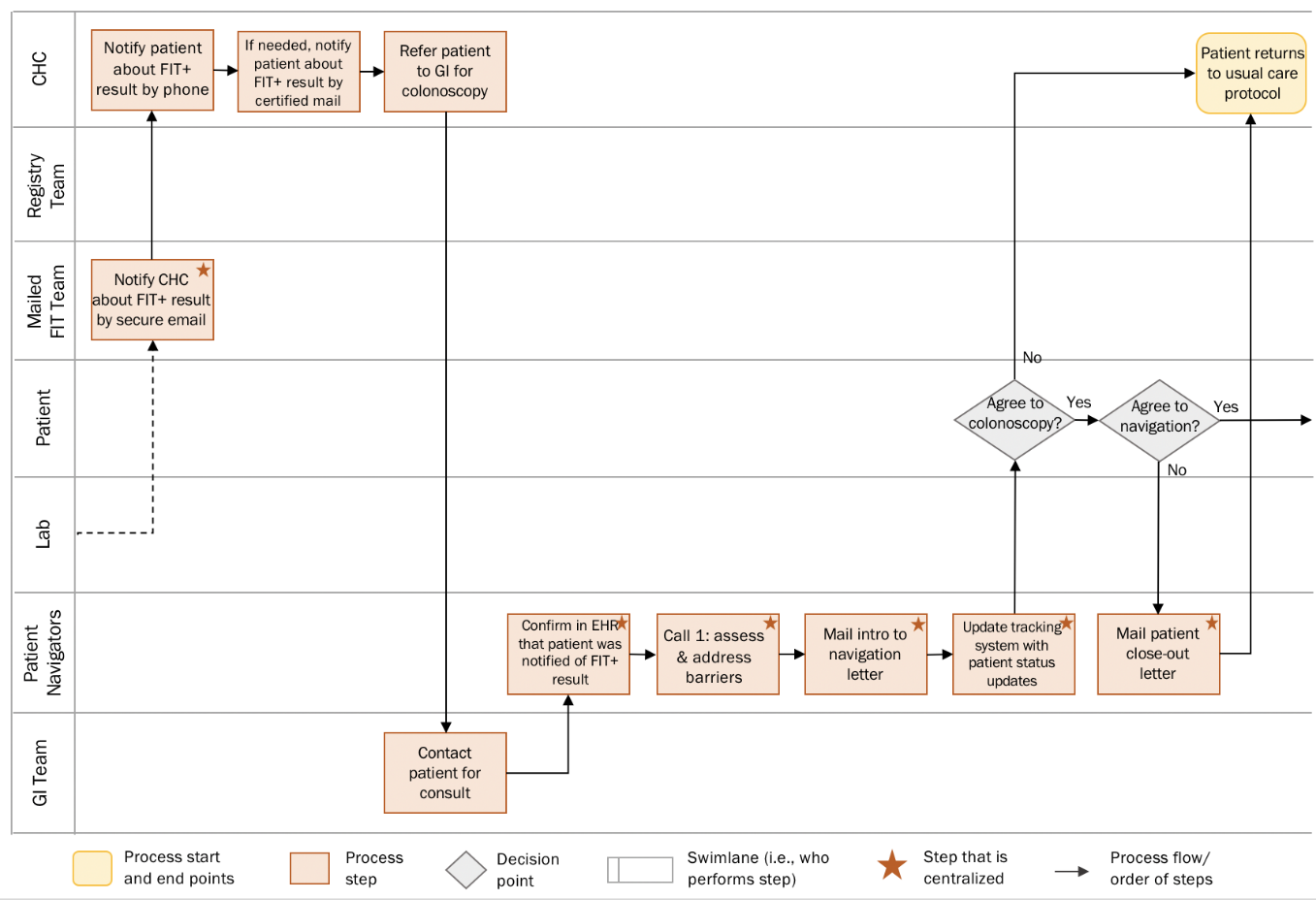

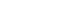

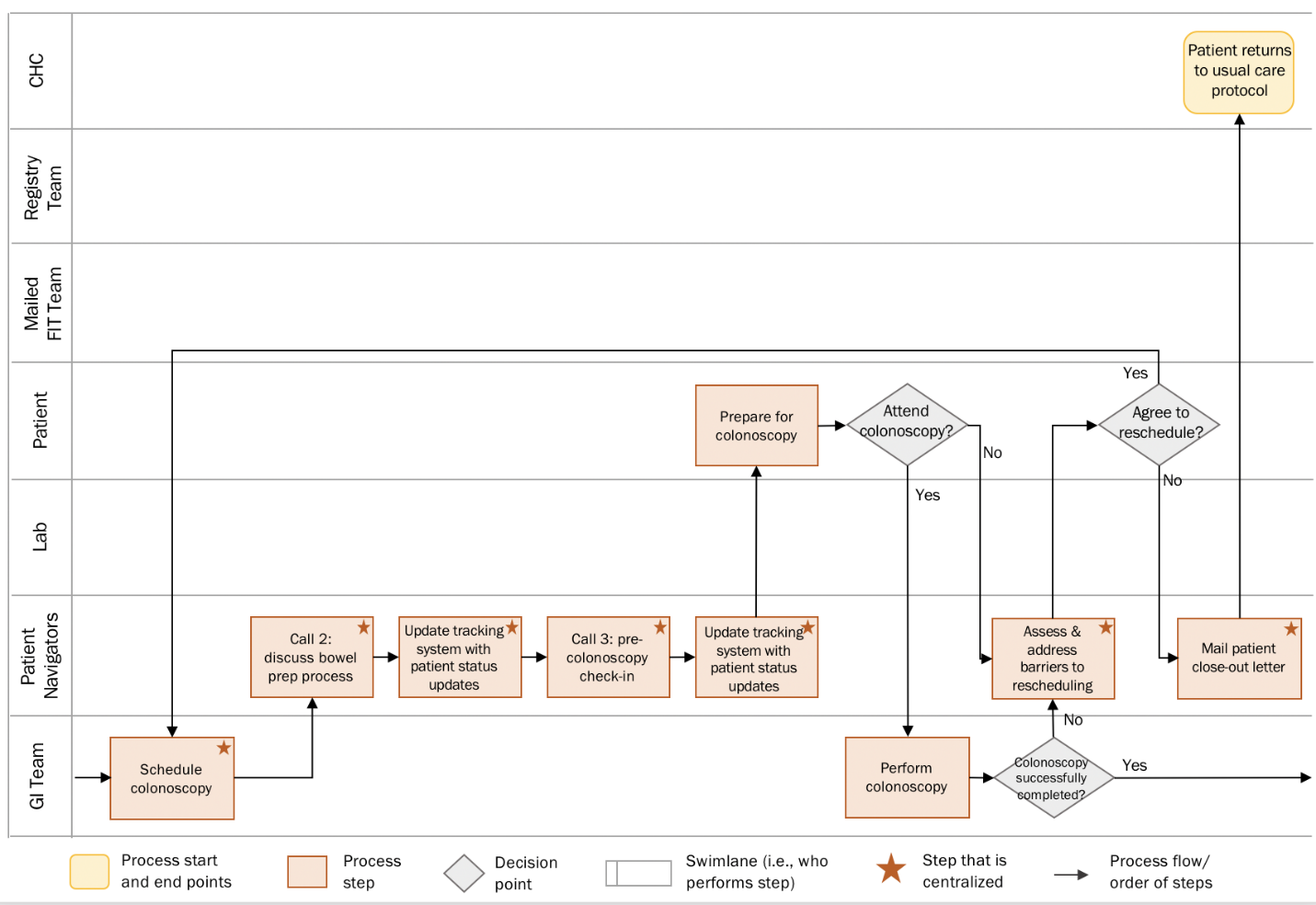

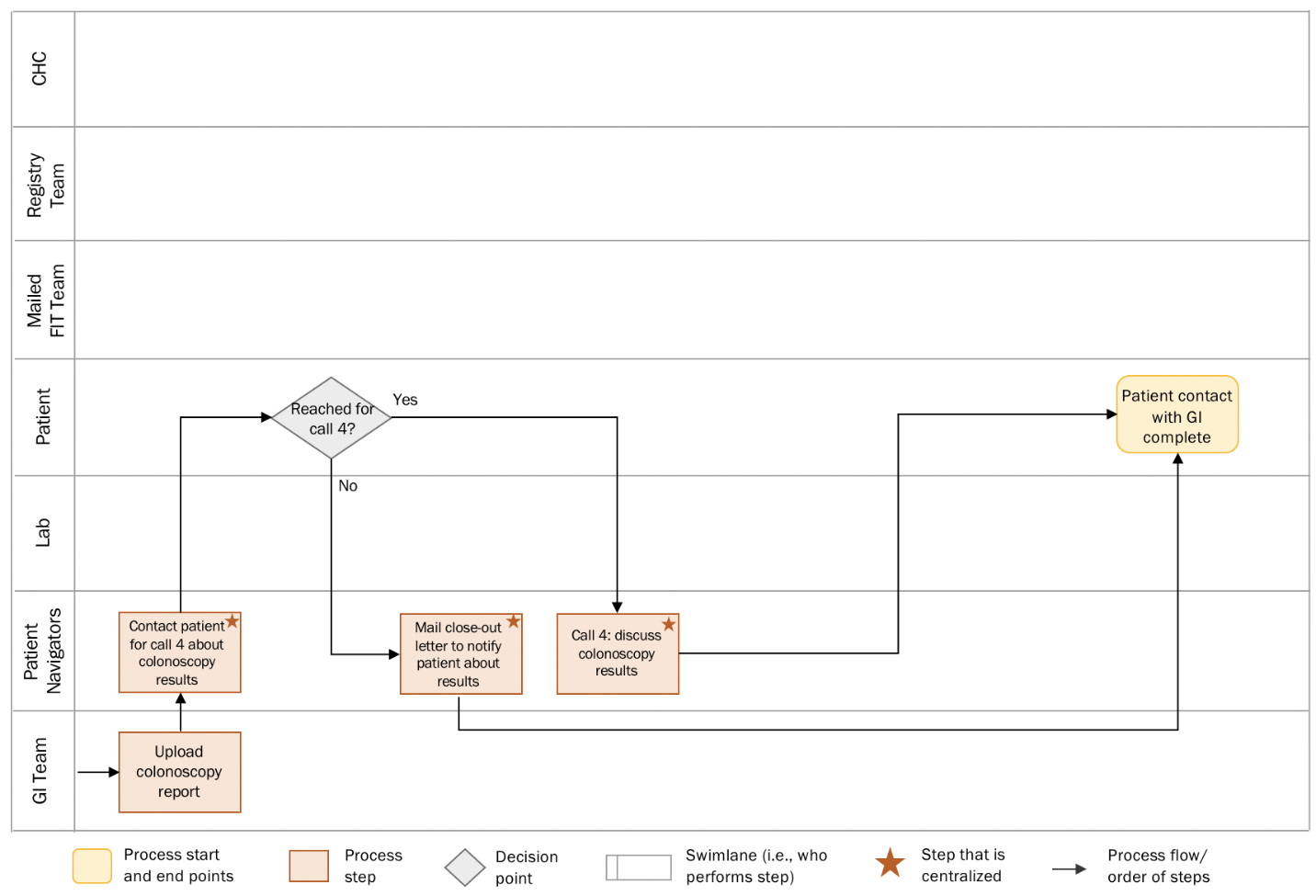

Supplement: Supplementary file 1 — Supplementary file1 (DOCX 3296 KB) [file 10552_2023_1769_MOESM1_ESM.docx]
